# Parental Attitudes to Risky Play and Children’s Independent Mobility: Public Health Implications for Children in Ireland

**DOI:** 10.3390/ijerph22071106

**Published:** 2025-07-14

**Authors:** Fiona Armstrong, Michael Joseph Barrett, David Gaul, Lorraine D’Arcy

**Affiliations:** 1 School of Global Business, Technological University Dublin, D15 YV78 Dublin, Ireland; david.gaul@tudublin.ie; 2 Paediatric Emergency Research and Innovation (PERI), Department of Paediatric Emergency Medicine, Children’s Health Ireland, D12 N512 Dublin, Ireland; michael.barrett@childrenshealthireland.ie; 3 Women’s and Children’s Health, School of Medicine, University College Dublin, D04 V1W8 Dublin, Ireland; 4 Sustainability Action Research Lead, The Clocktower Grangegorman, Technological University Dublin, D01 K822 Dublin, Ireland; lorraine.darcy@tudublin.ie

**Keywords:** risky play, parents, outdoor play, child safety

## Abstract

Background: Understanding the determinants of children’s outdoor play is an important element for child development and broader public health outcomes. There is growing evidence that children’s opportunities for play, particularly outdoor risky play, are diminishing. Parents are concerned with keeping their child safe while affording them independence to play. This study explored parents’ attitudes to risky play and practices around children’s independent mobility in Ireland with the aim of informing public health strategies promoting healthy childhood environments. Methods: An online survey comprising validated scales and standardised questions was completed by a nationally represented sample of 376 parents of children up to 16 years. Data was analysed via descriptive statistics, chi-square tests, and regression analysis. Results: A total of 376 participants accessed the survey, of which 349 completed it. A total of 84% of participants were female. A total of 74% agreed that children need regular exposure to actual risk to develop risk management skills, and 71% trusted their children to play safely. Chi-square tests reveal significant associations between outdoor play in the rain and school travel (*p* < 0.01), and appropriate age to begin activities at home and in educational settings (*p* < 0.05). A moderate association was found between the method of school travel and children’s permission to play in the rain (Cramer’s V = 0.51). Respondents considered supervision to be a necessity to ensure their children’s safety. Overall, the results indicate that parents were risk-averse in three of the six categories of risky play, namely, play near dangerous elements, play with adult tools, and out-of-sight play. Conclusions: This study presents a descriptive analysis of findings from the Ireland State of Play Survey. Findings indicate that although parents recognise the benefits of risky play, there is some contradiction between parental attitudes and actual practices, with a lack of willingness or confidence in permitting their children to participate in all such activities.

## 1. Introduction

Playing is an instinctive and universal human behaviour providing opportunities for children to develop skills and learn about their individual strengths and limitations. Play is recognised as being vital for children’s healthy emotional, social, and intellectual development [1] and is protected in the United Nations Convention on the Rights of the Child [2]. General Comment No. 17 [3] reaffirms the fundamental right of every child to rest and leisure, to engage in play and recreational activities appropriate to their age, and to participate freely in cultural life and the arts. This comment calls upon state parties to take all appropriate measures to fully realise Article 31, as play is instrumental in child development. When children are free to play, they play naturally at the ever-advancing edges of their mental or physical abilities [4], learning to overcome challenges and pushing boundaries to develop their skills. Free play is how children practice and acquire the physical and intellectual skills required to be successful in their culture [4]. Outdoor play and play in nature are associated with health benefits, facilitating children to explore age-appropriate risks and challenges, increasing awareness of their abilities, and fostering a sense of autonomy [5,6,7]. Although play in nature is appealing, weather conditions can act as barriers to outdoor play [8,9,10,11,12]. Risky play, synonymous with adventurous play, is a form of physical play involving uncertainty and a risk of physical injury [6], mainly occurs outdoors, where children value opportunities for risk and challenge in their play [13]. The benefits of risky play include increased physical activity, reduced sedentary behaviours, developing tolerance of uncertainty, increased social competence, and resilience [14]. Risk in the context of risky play denotes a situation whereby a child can recognise and evaluate a challenge and decide on a course of action [14]. Therefore, from a public health perspective, outdoor risky play is associated with significant health benefits [14]. Despite evidence supporting risky play, there has been growing awareness that children have increasingly fewer opportunities for play in urban spaces, in educational settings, and in the family home [1], with this decline of opportunities for play being linked to urbanisation, risk aversion in parents, and tensions within education systems [1,15]. Parental perceptions influence children’s interests and curiosities towards nature and the outdoors [16,17,18], and studies show that parents have fears and concerns regarding their child’s safety while playing outdoors [8,19]. Limiting children’s exposure to risk reduces their opportunity to learn effective skills, thus impeding their physical development [20] (p. 121). As such, the role of parents is important, as they can allow or restrict access to play activities and intervene to stop the play [21].

### 1.1. Risk-Averse Society

Western high-income society is becoming increasingly risk averse, impacting the opportunities for free and risky play [22,23,24]. Risk perception has been shown to be culturally specific [25,26,27], and fear of injury is a factor in parents’ choice of play opportunities for their children [22,28,29]). In Western societies, parents have become more involved in their children’s lives and the risks they are exposed to [30] and less willing for their children to take risks [31]. Similarly, China has a significant adult presence when children are playing outdoors [12] with parents and/or grandparents filling the role of play friends and caregivers. Time for outdoor play in China is also limited by the academic pressure on younger children, which is influenced by the one-child policy [32], and by reduced space available for play due to rapid urbanisation over the last seventy years [12]. Risky play benefits children’s health and development [13,22,33] through allowing them the freedom to develop skills for present and future challenges [13,34]. Rance, Ramchandani, and Hesketh [35] suggest that society’s tolerance of minor injuries in children has shifted from an understanding that bumps and bruises are a common element of childhood to something that must be prevented, resulting in children being kept as safe as possible and excluded from learning how to assess risk for themselves.

### 1.2. Child Safety Concerns

Increasing societal fears about child safety have heightened parental concerns, resulting in restricting outdoor play [4,36,37]. Children are now considered to require supervision and protection and to be less responsible and resilient [4] despite children in the Western world having high levels of personal safety [38,39]. Safety is considered inversely related to risk with limited definition or evidence in relation to children [40]. Child safety refers to a specific type of safety awareness focused on the unique hazards that children may encounter, such as taking precautions against dangers such as fire and exercising extra caution when crossing the road. Children naturally seek opportunities to challenge themselves, pushing boundaries and developing skills as they explore the world, and this process inevitably involves some level of risk [41]. Attitudes to play and safety vary across countries; however, concerns about play and safety are shared by parents worldwide [34,42,43,44,45], with policy and practice prioritising risk aversion [40,46]. Parents believe play should be safe, should contribute to learning, and should match a child’s competence [44]. Children’s freedom to move around their cities and neighbourhoods on their own without adult supervision has potentially impacted the opportunities for children to engage in outdoor play [47,48,49]. Parental attitudes influence children’s play and children’s independent mobility, thus impacting children’s opportunities to participate in risky play. Parents willingness to encourage their children to participate in risky play is influenced by positive attitudes to risk-taking [22,37,50].

### 1.3. International Support for Play

The importance of play to children’s health and wellbeing is recognised worldwide, with nations creating policies and frameworks to enhance children’s opportunities for free outdoor play and to address barriers. In Canada, two position statements on play advocate that health care providers prescribe outdoor free play as health advice [33,51]. Australia, while valuing play for healthy development and noting a decline in outdoor physical play, has a Play Today national campaign to get all children in Australia playing freely outside every day for better health and wellbeing. China, with recent research on play, currently has no policy framework, as is the case with most other countries in Asia, Africa, and South America. The New Zealand State of Play survey [29] found that parents considered exposure to risk to be beneficial for their children; however, parents considered supervision to be necessary for safety [29]. The free-range kids’ movement in the United States of America (USA) promotes children’s movement through public spaces without adult supervision [52], as independent mobility supports the development of motor, spatial, and navigation skills and cognitive function [53]. The Scandinavian countries, including Norway, Sweden, and Denmark, consider being connected to nature part of their culture, and outdoor play is therefore a necessity [19]. The British Isles, including, Scotland, England, and Wales, have policies and play sufficiency duty requiring authorities to assess the sufficiency of opportunities for play in their area, supporting children’s health and wellbeing. Northern Ireland (NI) has developed play policies, which has increased awareness amongst statutory, community, and educational service providers. Ireland, with its play policy and vision for being a country where the importance of play is recognised and where children can experience a range of quality play opportunities to enrich their childhood, supported through a national policy framework for children and young people and by local government and stakeholders actively engaged in improving play opportunities in each local environment. The play policy emphasises children’s right to safe, varied, and accessible play, which includes opportunities for risk-oriented activities. The interaction and partnering of statutory, community, and private sectors indirectly contributes to increasing parental confidence in allowing children to participate in unstructured and adventurous forms of play.

### 1.4. Children’s Independent Mobility

Children’s independent mobility considers children’s freedom for independent action, exploration, play, and socialising with friends in their local environments without adult supervision [54]. Ireland ranks 12th out of 16 European countries for children’s independent mobility, placing it among the lowest in Europe [55], with barriers identified as distance, urban or rural location, and general safety issues [56]. Unsupervised play in local neighbourhoods has declined in Western societies despite the opportunities it provides for skill development in the areas of social skills [57], traffic safety skills, and navigating public spaces competently [58]. Children’s permissions for independent mobility increase between the ages of 8 and 12 years and are influenced by gender, urbanisation, and socio-economic factors [26,59]. The New Zealand state of play survey results regarding independent mobility indicated that 13 years was the age children were allowed out unsupervised with friends, and 15 years was the most common age children were allowed out alone, while a similar study in the UK reported that on average children were allowed out alone around 11 years [22].

### 1.5. Societal Factors Influencing Opportunities for Play

Opportunities for play have reduced over the last fifty years, influenced by factors including an increased focus on formal education from a younger age, dual-income households reducing time available for play, urbanisation reducing the space to play, the quality, and accessibility of the play areas, and a decline in children’s independent mobility [14,24,60,61,62]. Government educational policy has also influenced opportunities for play, creating a focus on academic achievement [63,64], which has contributed to reduced children’s time for play. Competitive sports, while encouraging physical activity and improving health and fitness, also impact the time available for children to play [47,51]. Children require space and time for play, along with the ability to access these spaces independently, as they develop physical and cognitive skills. This independence is fostered as parents gain confidence in their child’s competence.

In summary, the benefits of children engaging in risky play are well established [6,9,34,65,66,67]. This has led to the development of numerous policy documents around the world, which support and encourage the independent play behaviours and opportunities for play for children. However, due to rapid urbanisation, reduction in green spaces, focus on earlier formal education, reduced independent mobility, and safety concerns, in recent years children’s ability to engage in independent play has been curtailed. Furthermore, increasing levels of risk aversion, changing parental attitudes to play, and the need for adult supervision have limited and sanitised play behaviours of children, thus reducing the benefits.

There are no studies to date on the attitudes of parents in Ireland to risky play. This study aims to investigate parental attitudes to risky play and the level of risky play allowed by parents, including play opportunities available to children and children’s independent mobility in Ireland. The research questions informing this study include what are Irish parents’ perceptions of the benefits and risks associated with different forms of risky play? What influences parents’ attitudes towards risky play and independent mobility, and how does this translate into actual autonomy granted to children in Ireland? How do parental beliefs regarding safety influence their decisions about children’s autonomy in outdoor spaces?

## 2. Methodology

### 2.1. Design

The target population was parents of children up to age sixteen years. The sample was a convenience sample, as this was an open survey.

### 2.2. Approval

The study received ethical approval from Children’s Health Ireland (REC-382-23) and Technological University Dublin (REIC-21-170).

### 2.3. Consent Process

Informed consent was achieved through each participant reading the Participant Information Leaflet (PIL) in advance of completing the online survey (Appendix A). The PIL explained the background to the study, the purpose of the study, the investigator and contact details, the process of completing the study survey, the length of time the survey would take, and data storage and protection information. No personal data was requested.

### 2.4. Development and Testing

The survey mimicked the validated New Zealand State of Play survey, comprising questions designed to determine parents’ perceptions and practices of risky play and independent mobility (Appendix B). The survey was created on the Microsoft Office Forms platform, which is visually and functionally similar to a paper-based questionnaire and could be completed on a personal computer or smartphone. Testing of the functionality of the electronic questionnaire was completed by a research group within TU Dublin to assess user friendliness, ensure data quality, ensure robust data collection, determine the time to complete the survey, and identify and address technical or usability issues in advance of launch.

### 2.5. Recruitment Process and Participants

Participants were recruited both through Children’s Health Ireland (CHI) hospitals, where participants were invited to complete the online survey by scanning a QR code available in all outpatient areas, and by online recruitment via social media from the Play and Risk in Kids (PARK) X platform. This was a voluntary survey. Participants were excluded from the survey analysis if they stated in the first question that they were under 18 years of age or not a parent or caregiver of a child under 16 years of age, were not living in Ireland, or if they did not complete the survey. Twenty-seven participants were excluded as they did not meet the inclusion criteria as outlined above.

### 2.6. Survey Administration

Participants completed the survey by clicking on the QR code and reading the PIL. Participants completed the online survey via the Microsoft Office Forms platform which is visually and functionally similar to a paper-based questionnaire. Microsoft Forms automatically collected the responses, and the data was available to the researchers only. Questions enquired about children’s age ranges, and parents without children in this age range were skipped through irrelevant sections of the survey. The survey took approximately 15 min to complete.

The survey was available online for 4 months during 2024, and there were no incentives offered. The 41 questions were distributed over approximately eight pages if using a desktop computer (more pages if completing on a smartphone). Participants could review and change their answer through a back button.

### 2.7. Materials

The State of Play survey comprises questions designed to determine parents’ perceptions and practices of risky play and independent mobility (Appendix B). The State of Play questionnaire from the New Zealand State of Play Survey was adapted for an Irish context, incorporating language modifications (removing the word bush) to align with Irish cultural nuances and incorporating questions from three questionnaires: the Tolerance for Risk in Play Scale (TRiPS), the Risk Engagement and Protection Survey (REPS), and an adapted version of the Perception of Positive Potentiality of Outdoor Autonomy for Children (PPOAC).

The Tolerance for Risk in Play Scale (TRiPS), developed to ascertain parents’ tolerance for children to experience risk during play, is a questionnaire enquiring if respondents allow their child to engage in activities pertaining to the six categories of risky play [68]. The survey asks parents to consider their responses to risky play scenarios with reference to their eldest child aged between 5 and 12. The TRiPS contains thirty items.

The Risk Engagement and Protection Survey (REPS) investigates the views and attitudes of parents towards protecting children from injury and allowing them to engage in risks [69]. Parents were asked to rate how much they agree or disagree with statements using a five-point Likert scale anchored with “strongly agree” and “strongly disagree”. The survey contains twelve items, six related to protection from injury and six related to risk engagement.

An adapted version of the Perception of Positive Potentiality of Outdoor Autonomy for Children (PPOAC) scale [70] was included in the survey to establish the extent parents believed roaming the neighbourhood was positive or negative for 9–10-year-olds, the age group the questionnaire has been validated in/with. There are nine items in the scale with answers on a Likert scale ranging from strongly agree to strongly disagree.

Risky play is defined as thrilling and exciting forms of physical play that involve uncertainty and a risk of physical injury [6]. Sandseter [71] identified six categories of risky play as play at great heights, play at rapid speed, play with dangerous tools, play with dangerous elements, rough and tumble play, and out-of-sight play or risk-of-getting-lost play (Appendix C). In consideration of potential barriers to risky play participation, participants were asked the extent they agreed or disagreed with statements regarding barriers to allowing children to engage in risky play. Responses were rated on a five-point Likert scale from “strongly agree” to “strongly disagree”. As per the New Zealand State of Play Survey, additional questions relating to playing in the rain were included in the survey addressing the frequency of play in the rain and reasons why children may not play in the rain. Participants were asked at which age, if at all, a child should first be allowed to engage in each category of risky play. This question was included to determine whether perceptions of risky play changed depending on a child’s age. Participants were then asked to indicate on a five-point Likert scale how often their eldest child engages in a selection of activities with varying degrees of supervision. The activities included examples from each of the six categories of risky play, as defined by Sandseter [71]. Questions also included those from a validated previous study on children’s travel behaviours and independent mobility [55,72] as children’s independent mobility is considered a subset of risky play. Participants were asked to answer yes/no or not applicable (N/A) to six items regarding travelling alone.

### 2.8. Statistical Analysis

This paper presents a statistical overview of a population sample of Irish households. Data was processed and analysed using Excel and IBM SPSS Statistics version 24, including functions such as descriptive statistics and graphical representations and creating visual data representations as charts and graphs.

Chi-square tests were used to analyse categorical data to determine if there is a statistically significant relationship between variables. Statistical significance was *p* < 0.05. Cramer’s V is used to measure the strength of association between two categorical variables. While specific guidelines for interpreting Cramer’s V values (e.g., weak, moderate, strong) can vary, a value of 0.1 to 0.3 is often considered a weak association, 0.3 to 0.5 a moderate association, and above 0.5 a strong association.

### 2.9. Limitations

This study is subject to limitations. Firstly, the use of quantitative data inherently restricts the depth of the responses. The survey design relied on closed-ended questions (e.g., likely, not likely), which limited the respondents’ ability to express mixed feelings and to elaborate on their perspective, which may have added further clarity to responses, providing contextual insights. Additionally, the sequential nature of the survey may have introduced response bias, as reading one question after another may have influenced participants thoughts and influenced their interpretation of the questions. There is a risk that respondents tailored their answers to reflect how they wish to be perceived, potentially compromising the authenticity of the data. Furthermore, the method of survey distribution, in a hospital waiting setting, online, and through social media platforms, may have influenced the demographic composition of the sample.

The sample consisted predominantly of female respondents, which may have introduced a gender bias in the findings. This limits the generalisability of the data to all parents, particularly in understanding parental attitudes.

## 3. Results

A total of 376 participants accessed the survey, of which 349 completed the survey, representing a mix of rural and urban geographical areas. Participant characteristics are outlined in Table 1. Most participants were mothers or grandmothers (84%) in the 45–49 age range (27%).

All children in the 9–10 age group were required to travel to school, with 40% using public transport for their commute (Table 2). Approximately 18% walked, while 17% travelled by bicycle or scooter. However, only 18% of children travelled to and from school independently (Table 2).

**Figure 1:**
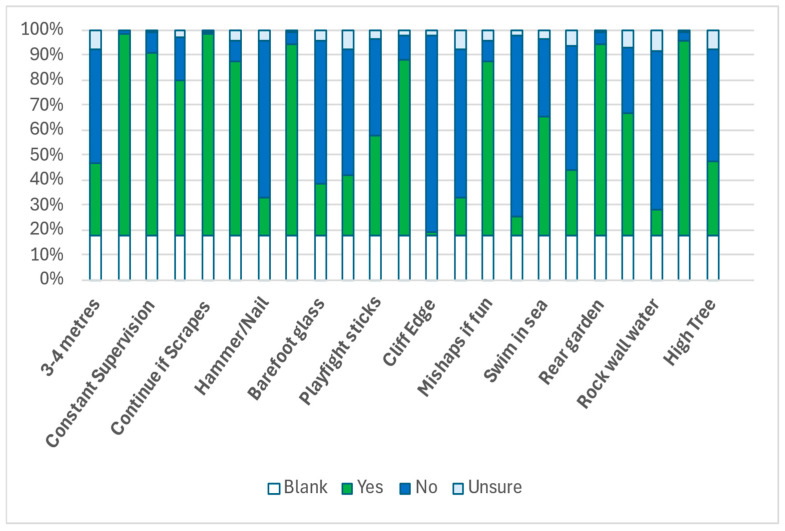
Risk Engagement and Protection Survey.

The majority of respondents had a positive perception of risk engagement (Figure 1). However, few participants were willing to allow their children to play near hazardous elements, such as at a cliff edge (1.5%), running close to an open fire (7.74%), or climbing a rock wall with a sheer drop into water (24.7%) (Figure 1). Participants largely did not allow their children to play out of sight in rural, undeveloped areas, highlighting parents’ need for supervision and the ability to intervene (Figure 1). This reluctance aligns with findings on independent mobility, as only 18% of children travelled to school alone (Figure 1).

Similarly, 62% of parents did not permit their children to use adult tools such as hammers and nails, despite safety guidelines recommending supervised use from age three (excluding power tools) (Figure 1). Power tools, on the other hand, are considered safe for children from age 14 with proper instruction and supervision (Figure 1).

Parents were risk-averse in three of the six categories of risky play, namely, play near dangerous elements, play with adult tools, and out-of-sight play.

**Figure 2:**
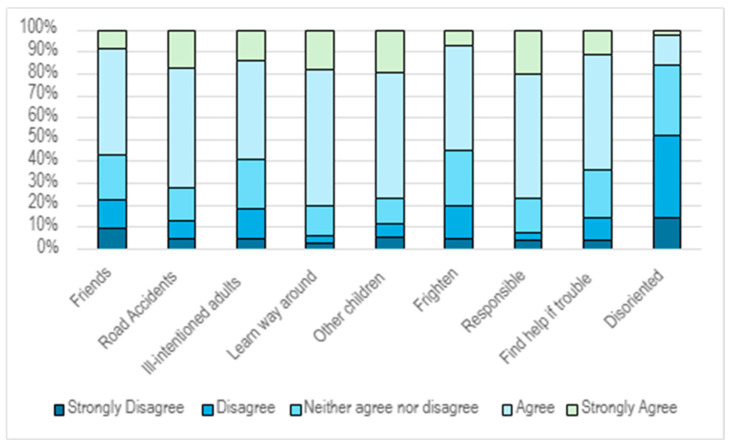
Perception of Positive Potentiality of Outdoor Autonomy for Children.

The perception of the Positive Potentiality of Outdoor Autonomy for Children (PPOAC) was positive, with participants viewing outdoor autonomy as beneficial for childhood development (Figure 2). There was no statistically significant difference between rural and urban parents’ responses. While many parents believed their children might encounter road accidents (72.2%) or see things that could frighten them (55%), a majority (63.6%) also felt confident that their child would be able to find help if needed (Figure 2). Parents’ trust in their child’s competence in various situations plays a key role in determining their willingness to allow independent mobility (Figure 2).

### Rain as a Potential Barrier to Risky Play

**Figure 3:**
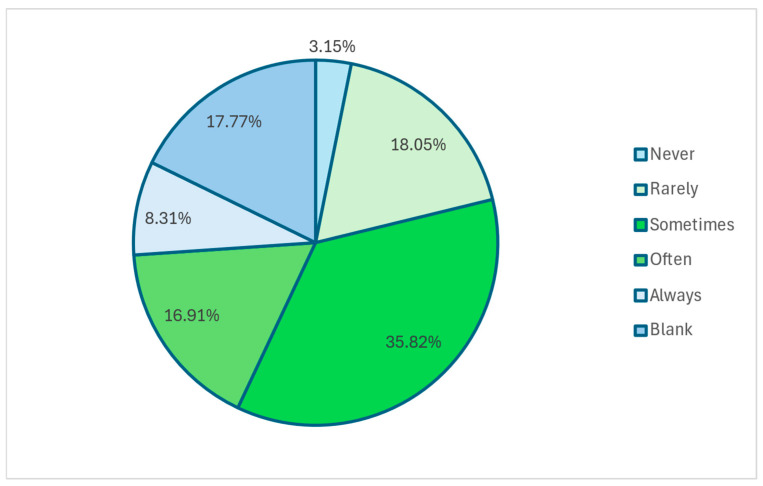
How often does my child play out in the rain?

The most common reasons identified for why children do not play in the rain were that ‘my child does not like being out in the rain’ (9.8%), ‘my child may get sick’ (4.9%), and ‘it would be too cold for my child’ (4.3%) (Figure 3).

Chi-square tests were used to analyse parental attitudes towards outdoor play in the rain and school travel (*p* < 0.01), as well as responses regarding the appropriate age for children to begin various activities at home and at school or early childhood learning centres (*p* < 0.05).

The analysis revealed that no significant correlation was found between location and parental attitudes towards children’s independent mobility. However, using Cramer’s V, a moderately strong statistical correlation (0.51) was identified between the method of school travel and respondents’ willingness to allow their children to play in the rain. Cramer’s V showed a strong statistical correlation (0.97) between the age at which activities are deemed appropriate and the setting in which they are conducted (home versus school or early childhood centre). Responses revealed notable age differences across activity categories, suggesting that the appropriateness of activities varies significantly by location.

**Figure 4:**
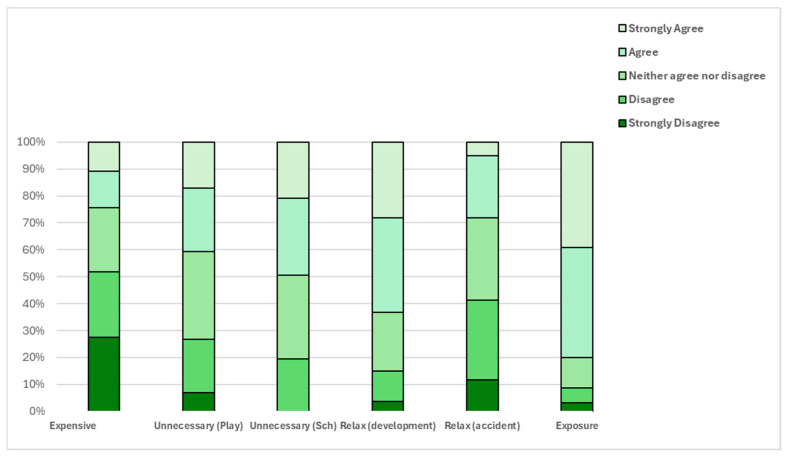
Tolerance for Risk in Play Scale measuring parental responses to risky play scenarios.

The responses to the statements in the TRiPS survey indicate that parents do not consider finding ways to get children active to be expensive (Figure 4). Parents also agree that children require exposure to develop risk management skills (Figure 4). Parents also consider allowance for activities to be age dependent (Figure 4).

## 4. Discussion

This study examined parental attitudes toward risky play and the play opportunities available to children, including aspects of independent mobility in Ireland. This is the first study in Ireland to explore parents’ perceptions of risky play and the extent to which such opportunities are available to children. A total of 74% of parents agreed that children need regular exposure to actual risk to develop risk management skills, and 70% of parents in the study trusted their children to play safely (Figure 4). However, despite this belief, many parents remained hesitant to allow certain types of risky play (Figure 4). This gap between beliefs and behaviours is relevant from a public health perspective, and the findings of this study reflect similar trends in New Zealand [29], Australia [60], Canada [28], and the UK [22], where parents are found to be aware of the benefits of risky play but choose not to allow their children to engage in such play.

Interestingly, while 70% of parents in this study trusted their child to play safely, the majority still restricted play near dangerous elements, with 63% not permitting play with adult tools, 63.3% restricting play near the water’s edge, and 78.5% prohibiting play at a cliff edge. This reveals some contradiction between parental attitudes and actual practices, reflecting findings from similar studies in Canada [50] and Australia [61], where parents express a desire to foster independence and competence in their children while simultaneously prioritising their safety. This caution may limit children’s opportunities to develop skills that mitigate risks in real-life settings.

Out-of-sight play was not allowed by 59% of parents who participated in this study, indicating parents’ preference to supervise their children to ensure safety. Urbanisation and its effect on parents’ perception of neighbourhood safety, coupled with fear of being judged as a parent, can result in less independent and risky outdoor play [25,37,47,67,73].

In this study, 75% of respondents reported that they do not allow their child to go out alone after dark, with no difference in responses based on the child’s gender. Children’s independent mobility is a subset of risky play, as it considers children’s freedom for independent action, exploration, play, and socialising with friends in their local environments without adult supervision [54]. Independent travel refers to activities such as walking or cycling to school, shops, friends’ houses, sports facilities, parks, and playgrounds [74] and is influenced by factors like the child’s age, gender, and competence [37]. Darkness is a major obstacle for children’s independent mobility [75] and restricts possibilities to be outdoors by signalling time to return home and influencing parent practices [58,76,77]. Seasonal changes in the hours of daylight impact children’s mobility [78], with children spending more time active outside in summer months [79]. The age and sex of a child also influence parental permissions for independent mobility [26,59], although not in this study. Independent mobility is essential for children to develop social interactions with peers, acquire spatial and traffic safety skills for navigating public spaces, and build maturity in decision-making [57,58]. Children acquire knowledge of and familiarity with their local area through experience, and restricting these opportunities potentially limits skill development [78]. Parental attitudes are also shaped by societal norms, with many perceiving children roaming alone as a sign of poor parenting [37,80], which introduces judgement from other parents influencing parental restrictions on children’s autonomy.

Previous research indicates that adults tend to restrict children’s independent travel and outdoor play to areas immediately surrounding their homes [47,81], reflecting parents’ preference for their children to play locally and be easily accessed. This study identified independent mobility factors, namely being out alone after dark was not allowed by 75% of parents, cycling alone was not allowed by 67% of parents, walking on main roads was not allowed by 75% of parents, walking alone was not allowed by 45% parents, and travelling on buses (non-school) was not allowed by 66% of parents, as barriers to independent mobility (Figure 2). This study aligns with existing research on children walking or cycling on main roads, with 68% of respondents reporting that they do not allow their child to engage in these activities unsupervised. Notably, responses were consistent regardless of geographical area.

The emergence of the Playing Out Initiative in the UK, where streets are closed to traffic for a set period to allow children to play safely, along with the Play Street initiative in Fingal County Council, Ireland, creates opportunities where children can play safely within minutes’ walking distance from their homes rather than having to be driven to a park or playground. This initiative is a parent- and resident-led movement restoring children’s freedom to play out in the streets and spaces where they live, for their health, happiness, and sense of belonging (Playing Out). This new initiative, in an Irish setting, supports families to facilitate outdoor play where urbanisation has reduced such opportunities and addresses parents’ safety concerns while allowing children to play outdoors in their neighbourhood. These structured council-supported play events influence parental risk perceptions, shifting from fear to trust, demonstrating that low-risk adventurous play is safe, and fostering sociability and a sense of agency.

In this study 66% of respondents would not let their child travel alone on public buses. Travelling to school without adult supervision can support children’s autonomy and improve both children’s and their parents’ perception of safety [82] Independent mobility supports children’s physical health and psychosocial wellbeing through improving their social interactions [26,83] and is an indicator of personal independence [84]. The parental barriers to independent mobility have been identified as built environment (residential density, walkability, bikeability), traffic safety, distance, and social support [85]. Foster [53] found parents’ perceptions of fearing that their children may have a traffic accident and the possible presence of “strangers” have been associated with restricting their children’s freedom [47]. Parents allow more independent mobility when they perceive the community as having more social cohesion, that is, a smaller area that is better connected and safer [86,87,88]. Parents who allow independent mobility to and from school also allow their children more independent mobility in their leisure activities. Barriers to using public transport include distance from school, safety concerns, and infrastructure, and Ireland is considered to have a car-centred traffic culture [89]. Children from rural areas of Ireland had fewer options for public transport and were more likely to be driven to school, becoming children of the ‘backseat generation’, playing less outdoors, and having more adult supervision [23]. This may be impacted by reduced availability of options for public transport in rural locations, as car ownership may be a necessity for those living in less urban areas because of transport inequity and poor accessibility to transport services [90,91,92]. Inadequate infrastructure can result in higher use of cars for transport in rural areas in Ireland [93]. These findings reflect broader public health challenges in Ireland, including car dependency, limited active transport options, and low rates of independent school travel.

There are differences in the age children are allowed to be independently mobile, as outside influences and social norms contribute to parental permissions for their children’s mobility outdoors [94], and parents, in an effort to protect their children from danger, such as motorised traffic and strangers, restrict independent mobility [23,26,81]. The majority (83%) of participants of this study were female (mothers), and women are more concerned with real and perceived risks than men [67,95] and more likely to protect the child from risks associated with independent mobility. This potentially underrepresents more risk-tolerant perspectives of fathers and grandfathers, thus overlooking how fathers’ attitudes influence children’s actual play behaviours and independent mobility.

Ireland experiences an average annual rainfall ranging from 700 mm to 1000 mm on the east coast to 1000 mm–1400 mm on the west coast [96]. With rain occurring on 170 to 263 days per year [97], it is a factor of daily life in Ireland, therefore, assessing parents’ willingness to allow their children to play in the rain is a key aspect of this study. The majority of parents (57%) reported they allowed their child to play in the rain sometimes, often, or always. The reasons given for not playing in the rain included the child did not like the rain (9.8%), the child may get sick (4.9%), or that it would be too cold (4.3%). Only 2.9% of parents reported that they never let their child play in the rain, with previous research noting children to be less active with increased precipitation [98,99].

This study found that parents did not consider that there were too many safety rules for play in school. The provision of space, time, and permission for freely chosen play is reinforced in the United Nations Convention on the Rights of the Child, General Comment 17, recommendations to schools [3]. In Ireland, education policy allocates outdoor breaktimes with teacher supervision to ensure safety [100]. However, previous research indicates professionals consider conflicting discourses on safety and protection regarding risky play [60,101] and sometimes constrain risky play because they fear injury and negative evaluations by other parents [60]. Teachers report that they value children’s freedom to choose their own play; however, their primary concern is to ensure safety in the schoolyard [60,102,103]. Teachers in Irish schoolyards prioritise children’s safety in response to parental expectations to prevent all accidents [104], as the absence of guidelines for play in schoolyards coupled with litigation fears results in teachers prioritising safety [60,102,105] and possibly restricting play.

In this study the majority of parents (71%) reported they trusted their child to play safely; however, a substantial number of parents restricted aspects of risky play. The parents in this study restricted out-of-sight play in rural areas, restricted play with tools such as hammers and nails (63%), and restricted play near natural elements such as cliff edges (78.5%). In total, 69.3% of parents agreed that they would allow their child to experience minor mishaps if the child was having fun, although a majority (49.3%) also said they would not allow play if there was a risk of breaking a bone.

### Strengths and Limitations

This study used questionnaires previously validated, and therefore our findings can be compared with other studies. This also adds to the consistency in how the questions are interpreted and the validity of the results.

The use of short surveys on mobile platforms can often limit open-ended responses and lead to a lack of depth to responses. It was not possible to establish a shared understanding with the participants. A limitation of online survey methodology is not all parents use the X (previously Twitter) platform or scan QR codes; therefore, only those with strong opinions or motivation may have responded. Parents who completed the survey while in a hospital waiting room may be more cautious about risk or anxious in the setting. Responding to the survey while in a health care setting may influence answers and lead to socially desirable responses. Also, using Likert scale responses is restrictive when attempting to understand participant opinions.

## 5. Conclusions

This study offers valuable insights into parental perceptions of risky play in Ireland. While parents recognise the benefits of risky play, they remain somewhat hesitant to allow their children to engage in all aspects of it. Parents indicated that children participated in three of the six categories of risky play. Reservations in allowing play with dangerous tools, play near dangerous elements, and out-of-sight play existed. Parents generally do not perceive an excessive number of safety rules in early learning and care centres or schools, which supports teachers in Irish schoolyards prioritising children’s safety in response to parental expectations to prevent all accidents. This study would suggest that parents consider constant supervision as a necessity to ensure the safety of children and that they choose to limit opportunities for aspects of risky play over teaching their children to use tools safely and to assess risks for themselves. This potentially has broader consequences as these children develop and mature into adolescence without exposure to learning opportunities to develop risk assessment and safety skills. However, the findings highlight significant limitations on children’s independent mobility, which do not appear to be influenced by geographical location. Future research could explore a more diverse parent population, as the majority of participants in this study were female. Such research may help inform and improve parental support for risky play opportunities. Additionally, further studies could examine the key barriers to children’s independent mobility as perceived by parents and guardians.

## Figures and Tables

**Table 1:** Participant characteristics; location demographics; children in household demographics.

Measures	Count	Percent
**Relationship to Child**		
Mother	292	83.43
Father	52	14.86
Grandmother	3	0.86
Grandfather	1	0.28
Guardian	1	0.29
Age Range		
Prefer not to say	62	17.77
25–29 years	3	0.86
30–34 years	7	2
35–39 years	51	14.61
40–44 years	97	27.79
45–49 years	102	29.23
50–54 years	19	5.44
55–59 years	4	1.15
60+ years	4	1.15
**Educational Attainment Level**		
Bachelor’s Degree	164	46.99
Completed Primary school	1	0.29
Completed Secondary school	15	4.3
University training	29	8.31
Apprenticeship/Diploma/Certificate	71	20.34
Postgraduate or higher	69	19.77
**Location Demographics**	**Count**	**Percent**
Other	64	18.34
Large City (more than 100,000 people)	97	27.79
Smaller city (30,000–100,000 people)	24	6.88
Town (1000–29,999 people)	96	27.51
Small town community or village (<1000 people)	25	7.16
Rural (not small town)	43	12.32
**Children in Household Demographics**	**Count**	**Percent**
**How many Children Live in your House?**		
**1**	27	19.2
**2**	202	49.57
**3**	36	25.21
**4**	20	6.02
**Children under 8**		
**Yes**	228	65.33
**No**	121	34.67
**Children 9–16 in the household**		
Yes	222	62.61
No	127	36.39

**Table 2:** Active transport and children’s independent mobility.

Table of Active Transport and Independent Mobility Data			
Measures:	Category:	Count:	Percent:
**School Travel**			
Method of travelling to school	Walk	63	18.05
	Car	92	26.36
	Bicycle/Scooter	17	4.87
	Bus/Public Transport	142	40.69
	Other	35	10.03
Method of travelling from school	Walk	62	17.77
	Car	93	26.65
	Bicycle/Scooter	24	6.88
	Bus/Public Transport	139	39.83
	Other	31	8.88
Person accompanying child to school	On their own	64	18.34
	Adult(s)	27	7.74
	Sibling(s)	202	57.90
	Friend(s)	36	10.32
	Other	20	5.73
**Person accompanying child from school**	On their own	62	17.77
	Adult(s)	18	5.16
	Sibling(s)	128	36.68
	Friend(s)	19	5.44
	Other	27	7.74
**Other Travel**			
Permitted to travel alone within walking distance	Yes	113	32.38
	No	157	44.99
	N/A	79	22.64
Permitted to cross main roads alone	Yes	133	38.11
	No	149	42.69
	N/A	67	19.20
Permitted to cycle on main roads alone	Yes	39	11.17
	No	237	67.91
	N/A	73	20.92
Permitted out alone after dark	Yes	18	5.16
	No	264	75.64
	N/A	67	19.20
Permitted to travel by bus alone (excluding school buses)	Yes	36	10.32
	No	232	66.48
	N/A	81	23.21

**Figure A1:**
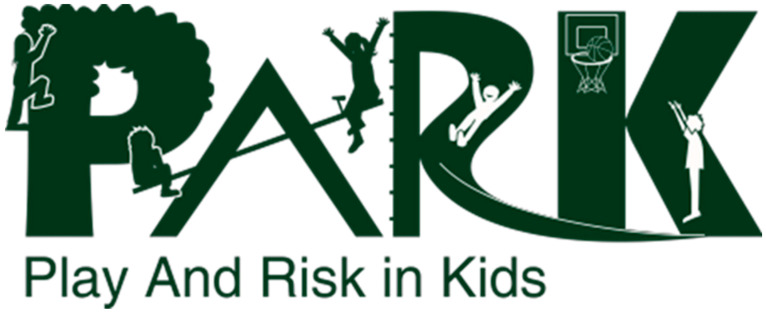
Play and risk in kids.

## Data Availability

Data is contained within the article.

## Appendix A. Participant Information Leaflet

**Name of Study:** An exploration of parental attitudes to risky play in Ireland.

You are being invited to take part in a research study that is being conducted by Fiona Armstrong and Dr David Gaul.

Before you decide whether or not you wish to take part, please read this information sheet carefully. Ask Fiona any questions. Don’t feel rushed or under pressure to make a quick decision. You should understand the risks and benefits of taking part in this study so that you can make a decision that is right for you.

**Why is this study being done?**

This study is part of the Play and Risk in Kids research project examining the current state of play infrastructure in Ireland, exploring parental attitudes towards risky play and investigating injury rates as a consequence of play in Ireland.

**Why have I been invited to take part?**

You are being invited to take part because you have children under the age of 16 and are living in Ireland. We are planning on interviewing parents of children aged between 0 and 16 years to learn about children’s opportunities for play and experiences of play in Ireland.

**Do I have to take part? Can I withdraw?**

Your participation is voluntary and should you decide not to consent there are no adverse consequences. You can withdraw your consent at any time up to one month following your interview and this can be done by emailing the researcher, Fiona on fiona.m.armstrong@mytudublin.ie.

**What happens if I change my mind?**

Participation in this study is voluntary and you may change your mind if you so wish and contact Fiona on email address fiona.m.armstrong@mytudublin.ie.

**How will the study be carried out and what will happen if I decide to take part?**

Participation in this study involves an online or telephone interview. There will be no face-to-face meeting. The interview will take approximately 60 min and you will be discussing your children’s play habits and opportunities, the time available for play, and the play choices that your children have. The researcher (Fiona) will contact you via email or phone (whichever you choose) to arrange a suitable time for the interview. The interview will be recorded for transcription, and you will have the opportunity to view the transcript approximately one week after the interview and before it is included in the study.

**Data protection**

The data from the study will be anonymised and stored on the cloud as per the Technological University of Dublin (TU Dublin) data protection policy which is GDPR compliant. The data will be password protected and stored on the cloud (Onedrive) and accessible only by Fiona Armstrong and Dr David Gaul (Supervisor). The data will be stored for five years as per the TU Dublin policy. No personal data will be used in this study and all data will be anonymised prior to data analysis.

**Are there any benefits or risks to taking part in this research?**

There is no direct benefit to taking part of this research study. There are no risks to taking part in this research study.

**Will I be told the outcome of the study?**

The results of the study will be reported in medical/scientific journals and disclosed at medical/scientific conferences. The data will be anonymised therefore no information which reveals your identity will be disclosed.

**Has this study been approved by a research ethics committee?**

Yes, this study has been approved by TU Dublin Research Ethics Committee. Approval was granted on 22 January 2024.

**Who is organising and funding this study?**

This study is funded as part of the Play and Risk in Kids project and as part of a PhD research project by Technological University Dublin.

**Is there any payment for taking part?**

No, there are no payments to participants for taking part in the study.

**Who should I contact for information or complaints?**

If you have any concerns or questions, you can contact:

Researcher: Fiona Armstrong at fiona.m.armstrong@mytudublin.ie

Principal Investigator: Dr David Gaul at david.gaul@tudublin.ie.

Under GDPR, if you are not satisfied with how your data is being processed, you have the right to lodge a complaint with the Office of the Data Protection Commission, 21 Fitzwilliam Square South, Dublin 2, Ireland.

## Appendix B. State of Play Survey PARK Project

This survey has been adapted from Jelleyman New Zealand State of Play Survey.

**Section 1**

Background of the study which Includes participant information leaflet and consent as per CHI template wording.

**Section 2**

**Household Structure (questions 2–11):**

How many children under age 16 live in your household? Answer 1 to 6 or more.

Do you have children aged 0–8 in your household? Yes/No

If yes then the age of the eldest male child in your household?

The age of the eldest female child in your household?

If No then do you have children aged 9–16 in your household?

If yes the age of the eldest male children age 9–16 in your household?

The age of the eldest female child age 9–16 in your household?

What is your relationship to the children? Drop down list.

What are the play opportunities available to your child/children? Please tick as many as relevant up to 9 options.

**Section 3**

**Perceptions of Play (questions 12–18) REPS**

What age if at all should children first be allowed to, the participant is asked to tick an age box for each of the following questions:

- Climb trees at home or in their local park/recreation areas
- Climb trees at their early childhood centre/school
- Engage in rough-and-tumble games (e.g., wrestling) at home
- Engage in rough-and-tumble games (e.g., wrestling) at their early childhood centre/school?
- Use adult tools (e.g., hammers, saws, drills) at home
- Use adult tools (e.g., hammers, saws, drills) at early childhood centre/school
- Roam their neighbourhood with friends but unsupervised by adults?
- Roam their neighbourhood alone?
- Roam their early childhood centre/school grounds during recess and lunch breaks unsupervised by teachers?
- Use loose parts (e.g., sticks, tires, timber) during outdoor play at home?
- Use loose parts (e.g., sticks, tires, timber, tarpaulins) during outdoor play at their early childhood centre/school?
- Engage in ‘messy’ play (e.g., mud, dirt, sand, water, paint) at home?
- Engage in ‘messy’ play (e.g., mud, dirt, sand, water, paint) at at their early childhood centre/school?
- Ride non-motorised vehicles (e.g., bikes, scooters, go-karts) in their neighbourhood while supervised by adults?
- Ride non-motorised vehicles (e.g., bikes, scooters, go-karts) in their neighbourhood with friends but unsupervised by adults?
- Ride non-motorised vehicles (e.g., bikes, scooters, go-karts) in their neighbourhood alone?
- Play on playground equipment (monkey bars, ladders, slides)?
- Play on trampolines?

Please indicate your level of agreement with the following 6 statements ranging from strongly disagree (1), disagree (2), neither agree nor disagree (3), agree (4), strongly agree (5).

- Finding ways to get children active is expensive these days.
- There are too many unnecessary safety rules applied to children’s play in Ireland today.
- There are too many unnecessary safety rules in Irish schools today.
- Relaxing the safety rules and introducing traditional risky play practices and equipment in schools would enhance children’s development.
- Relaxing the safety rules and introducing traditional risky play practices and equipment in schools would result in an increase in serious accidents and injuries.
- Children require regular exposure to actual risk in order to develop risk management skills.

**Section 4**

**Statements (question 19) PPOAC**

Please express your opinion about 9- or 10-year-old boys or girls who go out alone in the area around your home. The scale ranges from strongly disagree, disagree, neutral, agree, strongly agree.

I believe that 9- or 10-year-old boys or girls who go out alone in the area around my house can:

- Make new friends.
- Be exposed to the risk of road accidents.
- Encounter ill-intentioned adults.
- Learn his or her way around.
- Meet and/or play with other children.
- See things that may frighten him or her.
- Become more responsible.
- Find someone willing to help them in case of trouble.
- Feel disoriented when among people.

**Section 5**

**Child Activities (questions 20–22) TRiPS**

This section refers to the rules you apply to your oldest dependent child aged 5–12 years living in your household, most nights of the week.

Do you have aged 5–12 years in your household? Answer Yes proceed to next question;

Answer No then skip to end of survey and submit your responses.

The question asks about 31 different scenarios as follows with answer option of yes/no/unsure:

Would you let the child:

- Jump down from a height of 3–4 m?
- Allow the child play chase with other children?
- Trust the child to play by themselves without constant supervision?
- Trust the child to go head first down a slippery slide?
- Allow the child to continue playing if they get a few scrapes during play?
- Let the child have lots of challenges when they play at home?
- Let the child use a hammer and a nail unsupervised?
- Climb up a tree within your reach?
- Walk barefoot across a floor after broken glass had been swept up?
- Walk on slippery rocks close to water?
- Let the child play fight other children with sticks?
- Encourage the child to try new things that involve some risk?
- Engage in rough and tumble play?
- Play near the edge of steep cliffs?
- Allow the child play in undeveloped rural areas out of sight?
- Let the child experience minor mishaps if what they are doing is lots of fun?
- Let the child run close to an open fire?
- Swim in the sea close to the shore while you were watching from the beach?
- Allow the child to continue playing if there potential they may break a bone?
- Allow the child play in the rear garden unsupervised?
- Allow the child play-fight testing who is the strongest?
- Allow the child to climb a rock wall that goes straight down to the water?
- Would you wait to see if the child can manage challenges on their own before getting involved?
- Let the child climb as high as they want to in trees?
- Allow the child to ride a bicycle downhill at speed?
- Would you trust the child to play safely?
- Allow the child to use a sharp knife?
- Allow the child to play in a back garden supervised?
- Allow the child to balance on a fallen tree more than 2 m above the ground?
- Encourage the child to take some risks if it means having fun during play?
- Allow the child to climb up a tree beyond your reach?

**Section 6**

**Activity Patterns (questions 23) TRiPS**

This section refers to the activity patterns of your oldest dependent child aged 5–12. The questions ask how often does your child participate in 10 activities with answers ranging from never, seldom, sometimes, often, not applicable. The questions are as follows:

- Use adult tools (saws, hammers, drills)?
- Climb trees?
- Engage in rough and tumble games?
- Roam their neighbourhood with friends but unsupervised?
- Roam their neighbourhood alone?
- Use loose parts (sticks, tyres, timber) when playing outdoors?
- Engage in messy play (dirt, mud, sand, water, paint)?
- Ride non-motorised vehicles (scooter, bike, balance bike, go-kart) in the neighbourhood with friends unsupervised by adults?
- Ride non-motorised vehicles (scooter, bike, balance bike, go-kart) in the neighbourhood alone?

**Section 7**

**Weekly Activities (Questions 24–27)**

Q How many days in the last 7 days, did your child play or practice organised sport?

Answer options are one, through to six days, each day or no days.

How many days in the last 7 days did your child participate in other organised or structured activities (e.g., dance, music or gym)?

Answer options are one, through to six days, each day or no days.

How often do you allow your child play outside when it is raining? Answer options never, rarely, sometimes, often or always.

Which of the following would be reasons you would decide not to allow your child play outside in the **rain**. You may choose more than one option from the following:

- It would be too cold for my child
- My child may get sick
- My child would get too messy
- My child may slip or have an accident.
- I don’t like being outside in the rain.
- My child doesn’t like being outside in the rain.
- Not having suitable weatherproof clothing for my child
- I do not have suitable weatherproof clothing.
- Other

**Section 8**

**Values (questions 27) PPOAC**

This section refers to the values you apply to your oldest dependent child aged 5–12 years living in your household, most nights of the week. Answer options are strongly disagree, agree, neutral, disagree, strongly disagree.

Please rank your feelings on each of the following statements:

- I am concerned about the things I cannot control that can physically injure my child
- Fewer injuries happen to children when parents plan ways to prevent them
- I am concerned about the potential hazards in my home.
- Children should play in the places where there is low risk of injury
- Good supervision of my child means knowing what my child is doing at all times.
- Letting my child engage in physical activities without supervision greatly increases their chance of injury
- It is important for my child to engage in physically challenging experiences.
- I like to let my child find his or her physical limits
- I value opportunities for my child to explore new environments
- The benefits of physical activity for my child outweigh the risk of experiencing minor injuries
- I prefer to teach my child how to manage risky situations rather than avoid them.
- Participating in challenging and potentially risky physical activities will help my child develop self-confidence
- It feels as if I am always driving my child to an organised activity or sport.
- The number of organised activities or sports my child participates in is a source of stress for the family.

**Section 9**

**Travel (questions 29–38)**

These questions refer to the travel patterns of your oldest dependent child, aged 5 to 12 living in your household most days of the week.

Does your child currently attend a preschool, primary school or secondary school? Yes or No.

How does your child usually travel to school? Walk/bus/car/bike or scooter/other

How does your child usually travel from school? Walk/bus/car/bike or scooter/other

Who does your child travel to school with? Alone/adult/sibling/friend/other

Who does your child travel from school with? Alone/adult/sibling/friend/other

When going to places other than school that are within walking distance, is your child allowed to travel alone? Yes/No/not applicable.

Is your child allowed to cross main roads alone?

Is your child allowed to cycle on main roads alone?

Is your child allowed to go out alone after dark?

Is your child allowed to travel on buses (other than school buses) alone?

**Section 10**

**Background Information (questions 39–41)**

Q39 What location best describes where you live? Large city/smaller city/town/small town community or village/rural/other

Q40 What is your age range? From <25 years to >60 years.

Q41 What is the highest educational level you have completed? Primary/secondary/apprenticeship/bachelor degree/postgraduate/other.

## Appendix C

**Table A1:** Sandseters Six Categories of Risky Play.

Category	Risk Involved	Sub-Categories
**Great Heights**	Danger of falling	Climbing, jumping, Balancing on high objects Hanging/swinging at great heights
**Great Speed**	Uncontrolled Speed and pace that could result in collision with something or someone	Swinging, sliding or sledging at high speed. Cycling, skating or skiing at high speed. Running uncontrollably
**Dangerous tools**	Can lead to injuries and wounds	Cutting tools, and strangling tools.
**Dangerous elements**	Where children can fall into or from something	Cliffs, deep water, fire pits
**Rough and tumble**	Where children can harm each other	Wrestling, fencing with sticks, play fighting.
**Disappear/get lost**	Where children can disappear from adult supervision and get lost alone.	Exploring alone, playing alone in unfamiliar environments.
